# Utility of a Histone Deacetylase Inhibitor (PXD101) for Thyroid Cancer Treatment

**DOI:** 10.1371/journal.pone.0077684

**Published:** 2013-10-14

**Authors:** Shu-Fu Lin, Jen-Der Lin, Ting-Chao Chou, Yu-Yao Huang, Richard J. Wong

**Affiliations:** 1 Department of Internal Medicine, Chang Gung Memorial Hospital, Chang Gung University, Taoyuan, Taiwan, ROC; 2 Laboratory of Preclinical Pharmacology Core, Memorial Sloan-Kettering Cancer Center, New York, New York, United States of America; 3 Department of Surgery, Memorial Sloan-Kettering Cancer Center, New York, New York, United States of America; Consiglio Nazionale delle Ricerche (CNR), Italy

## Abstract

**Background:**

We evaluated the therapeutic effects of the histone deacetylase inhibitor PXD101 alone and in combination with conventional chemotherapy in treating thyroid cancer.

**Methodology/Principal Findings:**

We studied eight cell lines from four types of thyroid cancer (papillary, follicular, anaplastic and medullary). The cytotoxicity of PXD101 alone and in combination with three conventional chemotherapeutic agents (doxorubicin, paclitaxel and docetaxel) was measured using LDH assay. Western blot assessed expression of acetylation of histone H3, histone H4 and tubulin, proteins associated with apoptosis, RAS/RAF/ERK and PI3K/AKT/mTOR signaling pathways, DNA damage and repair. Apoptosis and intracellular reactive oxygen species (ROS) were measured by flow cytometry. Mice bearing flank anaplastic thyroid cancers (ATC) were daily treated with intraperitoneal injection of PXD101 for 5 days per week. PXD101 effectively inhibited thyroid cancer cell proliferation in a dose-dependent manner. PXD101 induced ROS accumulation and inhibited RAS/RAF/ERK and PI3K/mTOR pathways in sensitive cells. Double-stranded DNA damage and apoptosis were induced by PXD101 in both sensitive and resistant cell lines. PXD101 retarded growth of 8505C ATC xenograft tumors with promising safety. Combination therapy of PXD101with doxorubicin and paclitaxel demonstrated synergistic effects against four ATC lines *in* *vitro*.

**Conclusions:**

PXD101 represses thyroid cancer proliferation and has synergistic effects in combination with doxorubicin and paclitaxel in treating ATC. These findings support clinical trials using PXD101 for patients with this dismal disease.

## Introduction

The incidence of thyroid cancer has increased over the past three decades worldwide [1]. Increased detection of small tumors (≤ 1 cm) accounts for half of the increase, although other etiologies for this increase remain to be determined [2,3]. The most common pathologic types of thyroid cancer originate from follicular (papillary, follicular, poorly differentiated and anaplastic cancer) and parafollicular thyroid cells (medullary cancer). Patients with well differentiated thyroid cancer (papillary and follicular cancer) usually have favorable prognosis. However, there is limited treatment for patients who develop metastatic and radioiodine-refractory thyroid cancer, which is often incurable [4]. ATC is a rare and typically fatal malignancy, with a median survival of only six months. Medullary thyroid cancer (MTC) accounts for about 4 % of thyroid malignancies in the USA in 2012. Though two kinase inhibitors vandetanib and cabozantinib improve progression-free survival of MTC and were approved by FDA recently, no curable therapies are available for metastatic MTC [5]. Overall, the mortality from thyroid cancer has been slightly increasing yearly since 1992 [1]. Novel therapies are needed to improve the outcomes of patients with refractory thyroid cancer.

Histone deacetylases (HDACs) remove acetyl groups from lysine residues in histone and non-histone substrates, including transcription factors and proteins controlling cell cycle, proliferation and apoptosis. HDACs are divided into 4 classes according to their homology to their yeast orthologues and as appear to be promising targets for cancer therapy [6-8]. Inhibition of HDACs using small molecules induces multiple biologic effects, including ROS accumulation, cell cycle arrest, DNA damage and apoptosis that can lead to cytotoxic effects. Malignant cells are considered to be more prone to HDAC inhibitors than benign cells [6]. FDA has approved two HDAC inhibitors suberoylanilide hydroxamic acid (SAHA) and depsipeptide in the treatment of cutaneous T cell lymphoma.

In thyroid samples, ATC has highest proportion of HDAC1 and 2 overexpression (80% and 92%), followed by papillary cancer (61% and 53%) and normal thyroid tissue (0% for both), suggesting HADC1 and 2 contribute to thyroid cancer tumorigenesis and dedifferentiation [9]. In line with these findings, Na^+^/I^-^ symporter expression and iodine accumulation could be induced by HDAC inhibitors in poorly differentiated and undifferentiated thyroid cancer [10,11]. In addition to promoting cell re-differentiation, HDAC inhibitors are able to induce apoptosis in thyroid cancer [12-14]. These findings suggest that HDAC inhibitors have the potential to treat thyroid cancer through inducing cancer cell re-differentiation and apoptosis. Furthermore, combination therapy of HDAC inhibitor with chemotherapy may enhance therapeutic efficacy against ATC [14-16]. Therefore, HDAC inhibitors are potential agents in the treatment of patients with refractory thyroid cancer.

PXD101 (belinostat) is a pan-HDAC inhibitor with potent cytotoxic effects against a variety of cancer types through inducing apoptosis, regardless of inheritant resistance of chemotherapy [17,18]. The combination of PXD101 with docetaxel showed beneficial effects in treating ovarian and prostate cancer cells [19,20]. Clinically, combined treatment of PXD101, paclitaxel and carboplatin resulted in partial response in patients with solid tumors in a phase I trial [21]. PXD101 in combination with doxorubicin also has favorable results in the treatment of soft tissue sarcoma in a phase I/II trial [22]. These data prompted us to explore the therapeutic efficacy of PXD101 in thyroid cancer. We also assessed the combination effects of PXD101 and chemotherapy in treating ATC, the most aggressive type of thyroid cancer.

## Results

### Cytotoxicity and acetylation of histones H3, histone H4 and tubulin by PXD101

**Figure 1:**
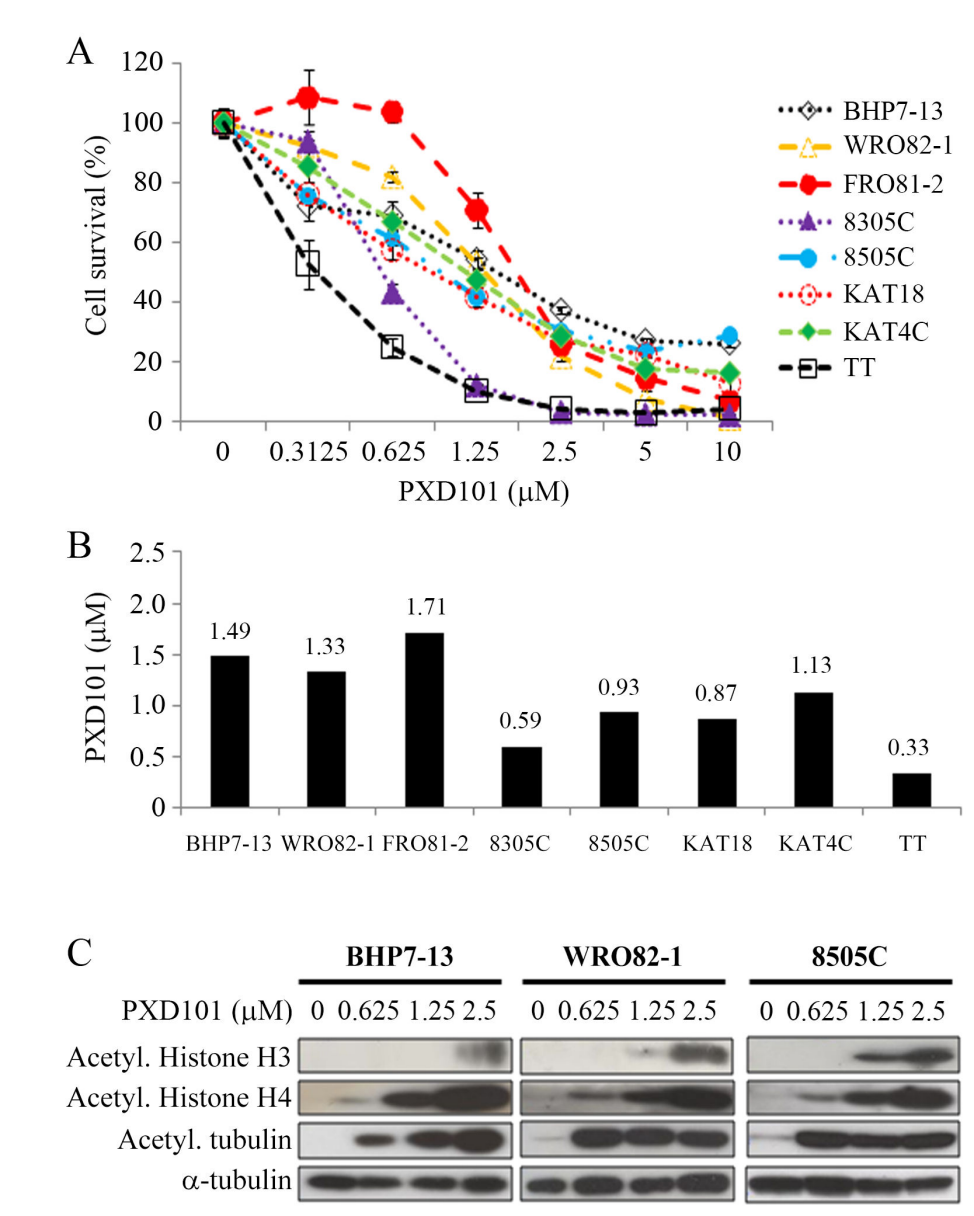
PXD101 induces cytotoxicity and increases acetylation of histone H3, histone H4 and tubulin in thyroid cancer cells. A, dose-response curves were obtained on day 4 from cells treated with a series of six 1:1 dilutions of PXD101. B, Dm of PXD101 on day 4 was calculated using CompuSyn software for each cell line. Among seven follicular cell-derived thyroid cancer lines, ATC cell lines (8305C, 8505C, KAT18 and KAT4C) had the lowest Dm, followed by well-differentiated follicular (WRO82-1) and papillary (BHP7-13) cancer, and follicular undifferentiated thyroid cancer (FRO81-2). Medullary thyroid cancer cells (TT) also had low Dm. C, PXD101 induced acetylation of histone H3 and histone H4 in a dose-dependent manner. PXD101 also increased acetylation of tubulin in BHP7-13, WRO82-1 and 8505C.

PXD101 inhibited cell proliferation in eight thyroid cancer lines in a dose-dependent manner (Figure 1A). These cell lines included a papillary (BHP7-13), a follicular (WRO82-1), a follicular undifferentiated (FRO81-2), four anaplastic (8305C, 8505C, KAT18, KAT4C) and a medullary (TT) human thyroid cancer. PXD101 at 1.25 μmol/L repressed at least 45% of cell growth in 7 of 8 cell lines on day 4. PXD101 at 10 μmol/L inhibited more than 70% cell growth in follicular cell-originating thyroid cancer lines and 95% in parafollicular medullary cancer (TT). The median effect dose (Dm) of PXD101 on day 4 was calculated for each cell line (Figure 1B). Among follicular cells-originating thyroid cancer, four ATC lines were more sensitive to PXD101 than follicular undifferentiated cancer and well-differentiated cancer (Dm; 8305C= 0.59 μmol/L, 8505C=0.93 μmol/L, KAT18= 0.87 μmol/L, KAT4C= 1.13 μmol/L, FRO81-2= 1.71 μmol/L, WRO82-1= 1.33 μmol/L, BHP7-13= 1.49 μmol/L). The Dm of MTC cancer line TT was 0.33 μmol/L. We explored the effects of PXD101 on three follicular cell-derived thyroid cancer lines in the following studies.

 Histone H3, histone H4 and tubulin can be acetylated by inhibition of HDACs. The effects of PXD101 to induce acetylation of these proteins were evaluated at 48 hours in 3 cell lines representing papillary (BHP7-13), follicular (WRO82-1) and anaplastic (8505C) thyroid cancer (Figure 1C). PXD101 ≥ 0.625 μmol/L significantly induced acetylation of histone H4 and tubulin in all cell lines. PXD101 ≥ 1.25 μmol/L increased acetylation of histone H3 in WRO82-1 and 8505C. In BHP7-13, acetylated histone H3 appeared when higher dose of PXD101 (2.5 μmol/L) was applied. These data suggests PXD101 is able to repress HDACs.

### Effects of PXD101 on apoptosis

**Figure 2:**
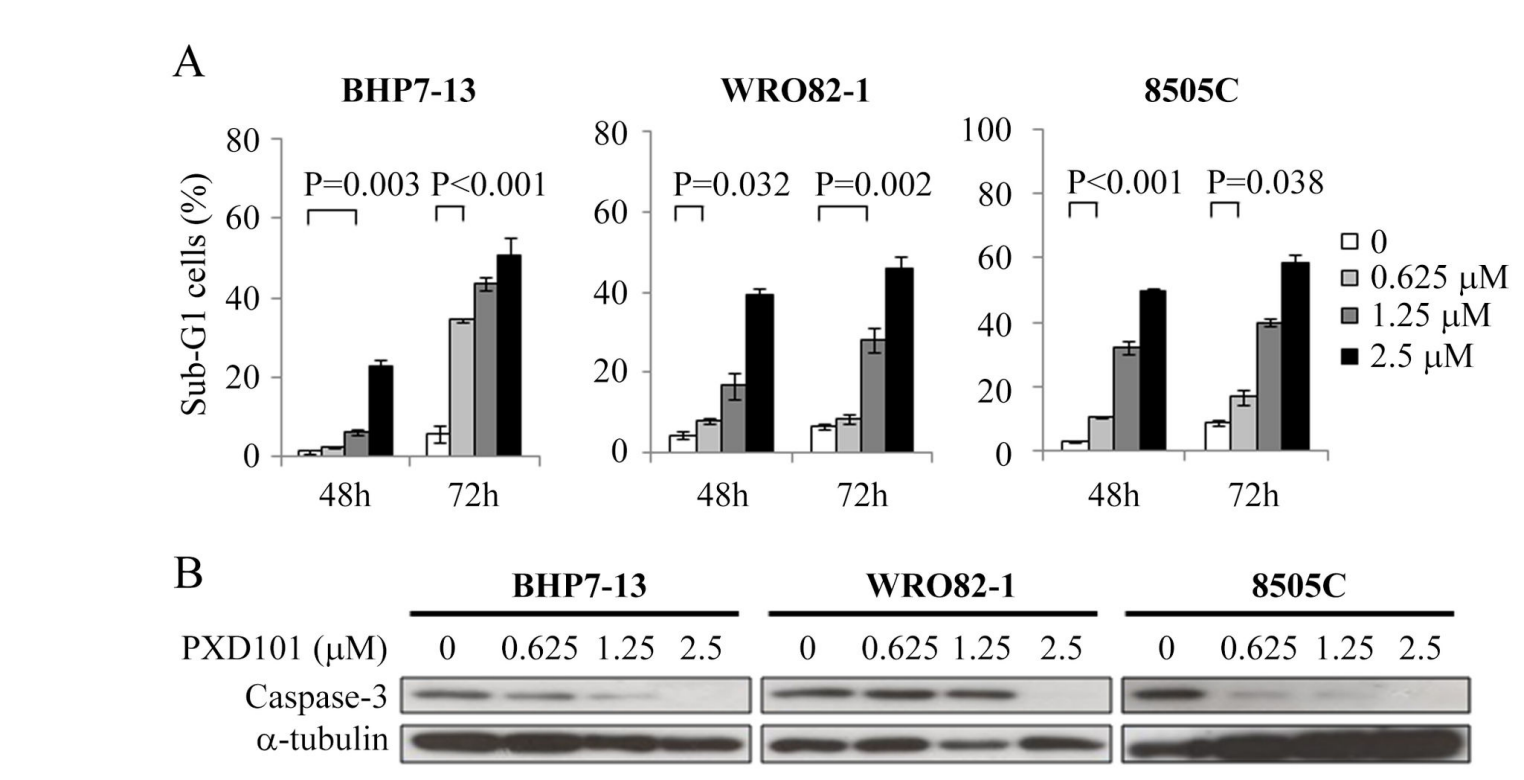
PXD101 induces apoptosis in BHP7-13, WRO82-1 and 8505C. A, apoptosis was analyzed by measurements of DNA content using flow cytometry at 48 and 72 hours. Increasing doses of PXD101 induced higher proportion of sub-G1 cells in all cell lines. B, immunoblot showed PXD101 degraded executioner caspase-3 in three cell lines.

BHP7-13, WRO82-1 and 8505C were exposed to PXD101 for 48 and 72 hours and sub-G1 cells were evaluated (Figure 2A). Compared with control, PXD101 at 1.25 and 2.5 μmol/L significantly induced apoptosis as determined by the proportion of sub-G1 cells at 48 and 72 hours in all cell lines.

To confirm the apoptotic effect, caspase-3 was evaluated by Western blot after 48 hours of treatment (Figure 2B). Higher doses of PXD101 caused more degradation of apoptotic executioner caspase-3 in BHP7-13 and 8505C. Degradation of caspase-3 was also observed in WRO82-1 when treated with PXD101 at 2.5 μmol/L. These findings supports apoptosis is the mechanism for the cytotoxicity of PXD101 in thyroid cancer cells.

### Effects of PXD101 on ROS accumulation

**Figure 3:**
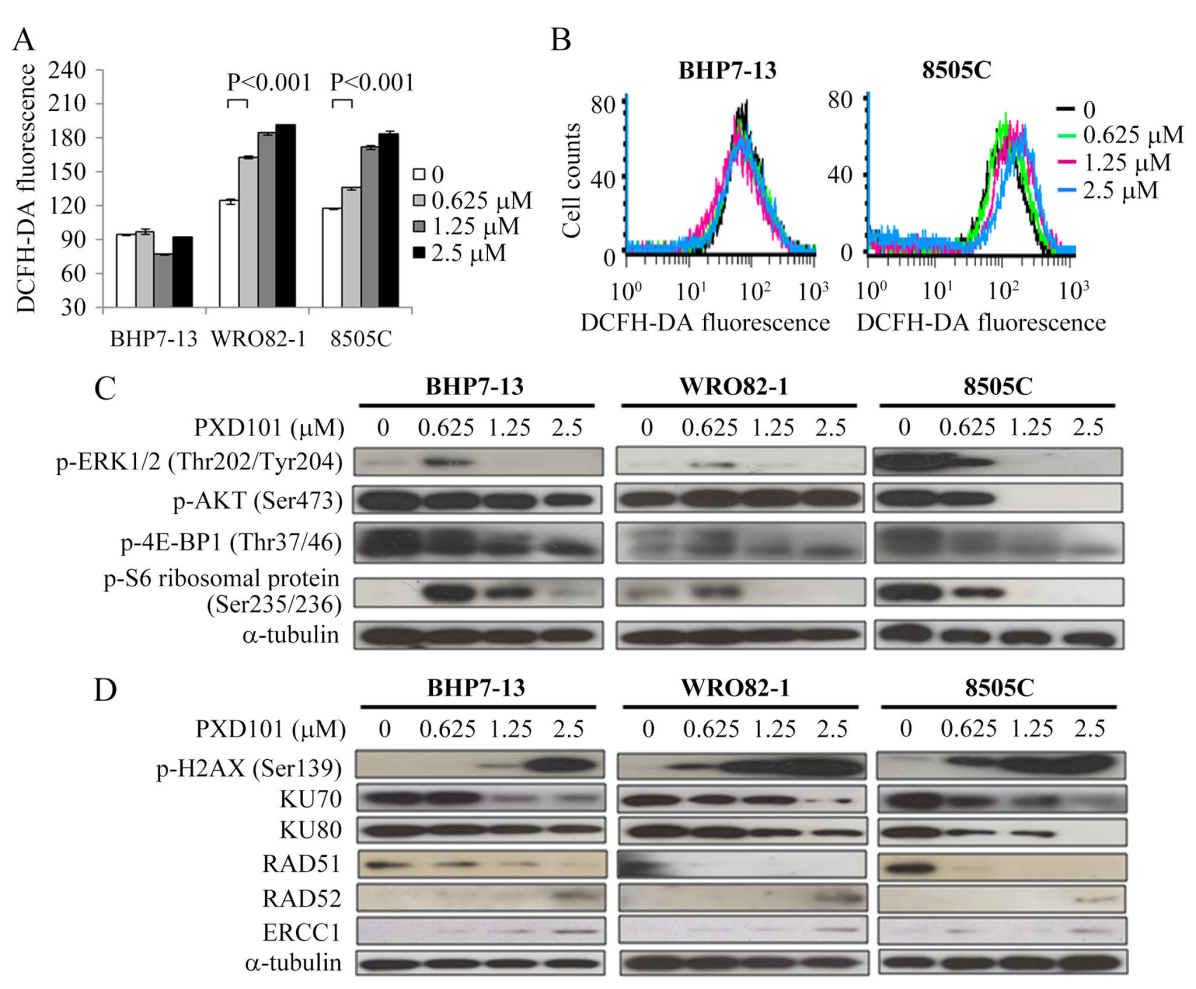
PXD101 accumulates ROS and inhibits RAS/RAF/ERK and PI3K/AKT/mTOR signaling pathways in sensitive cells, and induces DNA damage in all cell lines. A, DCFH-DA fluorescence was used to measure ROS by flow cytometry in thyroid cancer cells treated with PXD101 for 16 hours. Increasing doses of PXD101 produced more ROS in WRO82-1 and 8505C, but this effect was not observed in BHP7-13. B, two cell lines represent the ability of PXD101 to accumulate ROS. C, Immunoblot shows PXD101 repressed p-ERK1/2 (Thr202/Tyr204), p-AKT (Ser473), p-4E-BP1 (Thr37/46) and p-S6 ribosomal protein (Ser235/236) in 8505C. The inhibitory effects were not observed when low dose of PXD101 (0.625 µmol/L) applied in BHP7-13 and WRO82-1. D, increasing doses of PXD101 enhanced degradation of KU70, KU80 and RAD51, and enhanced expression of p-H2AX (Ser139), RAD52 and ERCC1 in BHP7-13, WRO82-1 and 8505C.

 Accumulation of ROS by HDAC inhibitors is considered one of the mechanisms accounts for cytotoxicity (6). We evaluated this effect by using 2′,7′-Dichlorofluorescin diacetate (DCFH-DA) assays to detect intracellular ROS in cells exposed to PXD101 for 16 hours (Figure 3A). Compared with control, PXD101 at 0.625 μmol/L significantly increased DCFH-DA fluorescence in WRO82-1 (163.1 ± 0.8 and 124.3 ± 2.4, P< 0.001) and 8505C (135.6 ± 0.9 and 117.1 ± 0.5, P< 0.001). Higher doses of PXD101 induced more ROS accumulation in these cells. However, this effect wasn’t observed in BHP7-13 which is less sensitive to PXD101. Longer exposure of PXD101 still failed to induce ROS accumulation in BHP7-13 (24 and 48 hours, data not shown). Two representative cell lines demonstrated the ability of PXD101 to induce ROS accumulation (Figure 3B). These findings suggest ROS accumulation can’t account for cytotoxicity of PXD101 in resistant thyroid cancer cell line.

### Modulation of protein associated with RAS/RAF/ERK and PI3K/AKT/mTOR signaling pathways by PXD101

RAS/RAF/ERK and PI3K/AKT/mTOR signaling transduction are important for the growth and survival of thyroid cancer (4). A prior report shows that a HDAC inhibitor can inhibit these pathways on leukemia cells [23]. We explored the effects of PXD101 on these pathways in BHP7-13, WRO82-1 and 8505C (Figure 3C). PXD101 consistently repressed p-ERK1/2 (Thr202/Tyr204), p-AKT (Ser473), p-4E-BP1 (Thr37/46) and p-S6 ribosomal protein (Ser235/236) in 8505C, which is the sensitive cell line. PXD101 had minimal effects on p-AKT in BHP7-13 and WRO82-1. Unexpectedly, low dose PXD101 (0.625 μmmol/L) activated p-ERK1/2 and p-S6 ribosomal protein in BHP7-13 and WRO82-1. p-4E-BP1 was also increased in WRO82-1. Therefore, PXD101 inhibits RAS/RAF/ERK and PI3K/AKT/mTOR signaling cascades in a sensitive cell line.

### Effects of PXD101 on DNA damage and repair

HDAC inhibitors are able to promote double-stranded DNA breaks (DSBs) and lead to apoptosis [24,25]. This mechanism may contribute to cytotoxicity of PXD101 in thyroid cancer. We evaluated the expression of proteins associated with DNA damage and repair in cells treated with PXD101 for 48 hours (Figure 3D). p-H2AX (Ser139), a traditional DSBs marker was significantly enhanced with a dose-dependent manner in all cell lines, supporting PXD101 can induce DSBs in thyroid cancer cells. Prior reports shows HDAC inhibitors can repress DNA repair proteins that contributes to DSBs [24-26]. We studied this possibility and found PXD101 considerably decreased a non-homologous end joining (NHEJ) DNA repair protein KU70 in all cell lines (Figure 3D). Another NHEJ protein KU80 was also repressed to some extent. Degradation of either KU70 or KU80 can lead to impairment of NHEJ repair. Homologous recombination (HR) repair is another important DSBs repair pathway. We proceeded to study the expression of a pivotal HR repair protein RAD51 and found RAD51 was notably repressed by PXD101 in all cell lines (Figure 3D). This data suggests that PXD101 can inhibit HR repair. When the pivotal DSBs repair machinery NHEJ and HR was compromised, the single-strand DNA annealing (SSA) pathway can be an alternative mechanism for DSBs repair [27]. This possibility was examined in this study as well, and the SSA proteins RAD52 and ERCC1 increased with exposure to PXD101 exposure in all cell lines (Figure 3D).

### Interaction of PXD101 and chemotherapy in ATC cells

The effect of combining PXD101 with different chemotherapeutic agents against ATC cells was evaluated. Three clinical relevant chemotherapeutic agents (doxorubicin, paclitaxel and docetaxel) were used for this study [28]. The Dm of these agents in each ATC cell line was reported previously [29]. Interactions between PXD101 and doxorubicin, paclitaxel and docetaxel were evaluated. The combination of PXD101 and each chemotherapeutic agent demonstrated favorable therapeutic effect in all ATC cancer lines (Figure S1).

**Figure 4:**
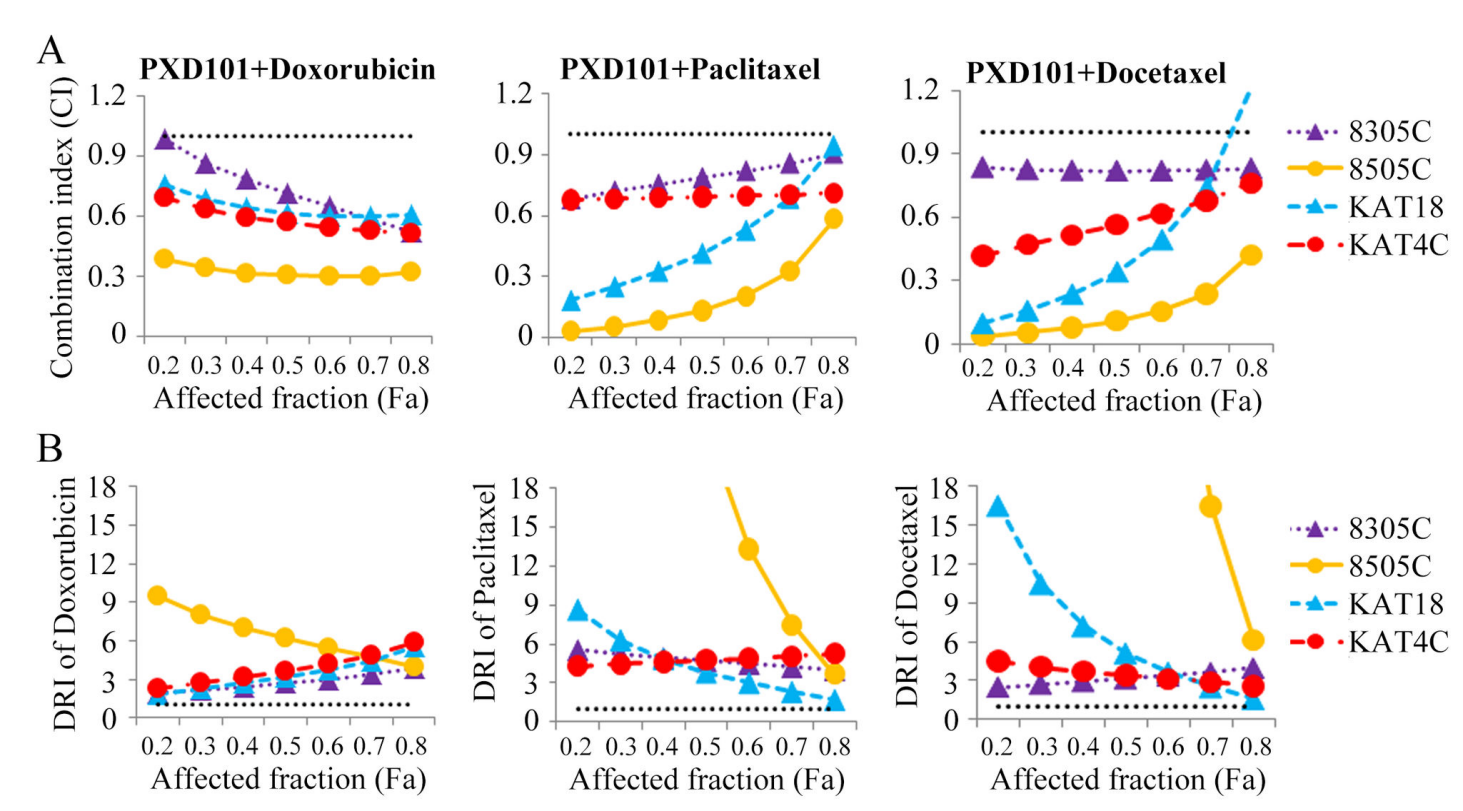
Combination treatments of PXD101 with doxorubicin and PXD101 with paclitaxel have synergistic effects against four ATC lines. A, CI of PXD101 with each chemotherapeutic agents was calculated using CompuSyn software for each ATC cell line. PXD101 plus doxorubicin and paclitaxel demonstrated synergistic effects in all cell lines, with CI < 0.8 at most conditions. PXD101 plus docetaxel was synergistic in 8305C, 8505C and KAT4C, and synergistic to antagonistic in KAT18. The horizontal dash lines at CI = 1 were drawn. B, the typical ranges of DRI values for chemotherapy in combination with PXD101. With the presence of PXD101, doxorubicin, paclitaxel and docetaxel all had favorable DRI. The lowest DRI of doxorubicin, paclitaxel and docetaxel all appeared in KAT18 (1.8, 1.5 and 1.6, respectively). The horizontal dash lines at DRI = 1 were drawn.

Interactions between PXD101 and chemotherapeutic agents were assessed by calculating the combination index (CI) by Chou-Talalay equation (Figure 4A), where CI < 1 indicates synergism, CI = 1 shows an addictive effect, and CI > 1 indicates antagonism [30,31]. Synergistic effects were recognized for the combination of PXD101 with doxorubicin and PXD101 with paclitaxel in all ATC lines (CI; 8305C= 0.52-0.98 and 0.68-0.91, 8505C = 0.32-0.39 and 0.03-0.58, KAT18= 0.61-0.76 and 0.18-0.94, KAT4C= 0.51-0.69 and 0.68-0.71, respectively). The combination of PXD101 with docetaxel were synergistic in 8305C, 8505C and KAT4C (CI; 8305C= 0.83-0.84, 8505C= 0.04-0.42, KAT4C= 0.42-0.76). The combination of PXD101 with docetaxel ranged from synergistic to antagonist in KAT18 (CI; 0.1-1.2). These results demonstrate that the combination of PXD101 with doxorubicin and paclitaxel has synergistic effects against all ATC lines, and the combination of PXD101 with docetaxel is synergistic in three of four lines. We calculated the dose reduction index (DRI) of each chemotherapeutic agent; the DRI indicates the fold of drug dose reduction in the presence of PXD101 (Figure 4B). Under exposure to PXD101, the doses of doxorubicin, paclitaxel and docetaxel can be reduced (all DRI > 1.0).

### PXD101 therapy of murine flank tumors

**Figure 5:**
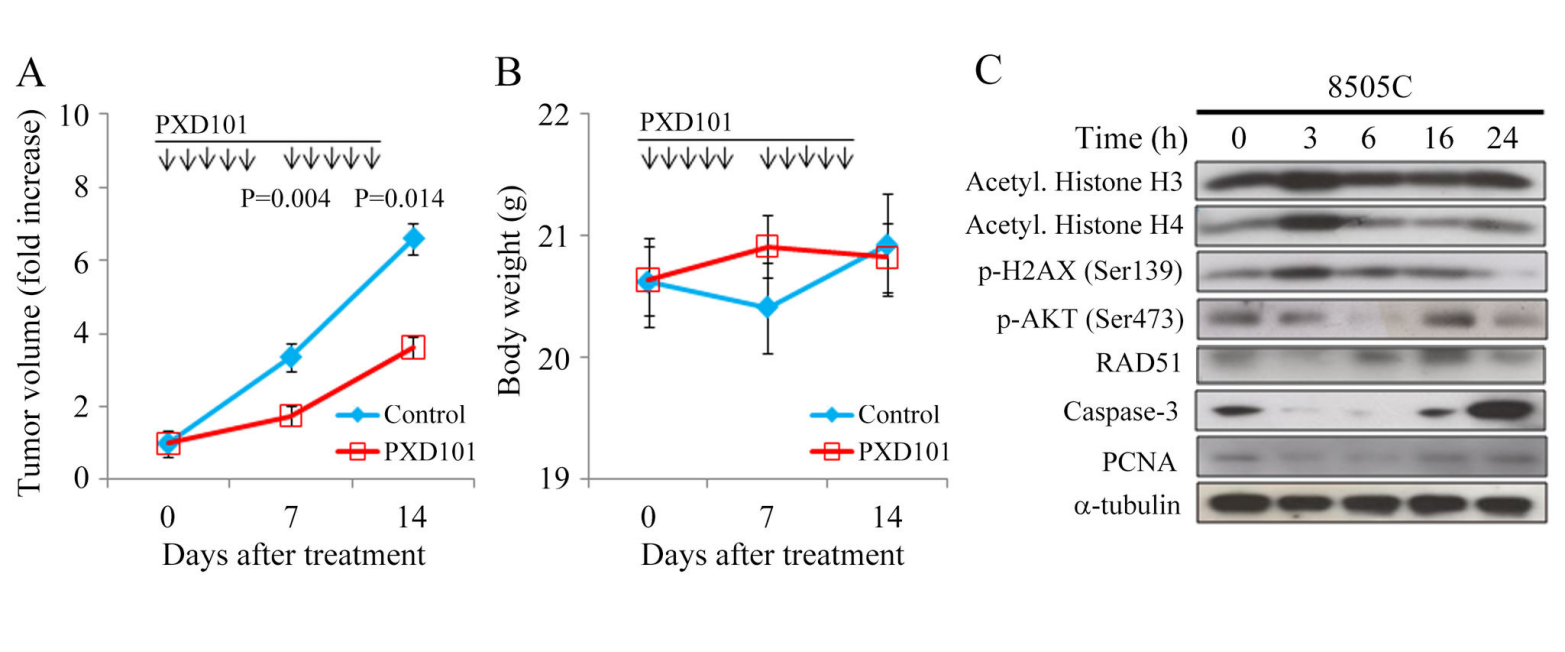
PXD101 induces acetylation of histones, causes DNA damage, promotes apoptosis, and inhibits the growth of ATC xenograft with promising safety in nude mice. A, daily intraperitoneal injection of PXD101 for 5 days per week repressed 8505C tumor growth. The differences of tumor volume increase between PXD101 and control group reached statistical significance on days 7 and 14. B, PXD101 didn’t cause significant weight loss in mice (P > 0.05). C, within 3 hours, PXD101 increased phosphorylation of H2AX and acetylation of histones H3 and H4, and repressed p-AKT, RAD51, caspase-3 and PCNA. The alterations of these proteins diminished by 6-16 hours.

The therapeutic effects and safety of PXD101 *in vivo* were evaluated in athymic nude mice bearing flank ATC 8505C and TT xenografts. Mice with established flank tumors were treated with intraperitoneal PXD101 (40 mg/kg) or vehicle daily for 5 doses per week until 14 days (Figure 5A). PXD101 significantly repressed 8505C tumor growth as compared to the control group on day 7 (1.7 ± 0.1-fold and 3.4 ± 0.5-fold, P = 0.004) and day 14 (3.6 ± 0.3-fold and 6.6 ± 1.0-fold, P = 0.014). PXD101 had no significant effects to change body weight during study period (Figure 5B). We did not observe any morbidity in these animals.

 The proteins that may be affected by PXD101 were evaluated in these animal tumors (Figure 5C). A single intraperitoneal injection of PXD101 (40 mg/kg) increased p-H2AX (Ser139) and acetylation of histone H3 and H4 by 3 hours and the effects diminished by 6 hours. RAD51 was repressed by 3 hours and it reappeared at 6 to 24 hours (Figure 5C, Figure S2). p-AKT (Ser473) was gradually repressed at 3 and 6 hour and this inhibitory effect was absent by 16 hours. The proliferation marker proliferating cell nuclear antigen (PCNA) was slightly reduced from 3 to 6 hours. PXD101 significantly degraded caspase-3 at 3 to 6 hours. These data reveal that PXD101 had robust but transient effects in ATC xenografts, and suggest that more frequent administration of PXD101 may enhance therapeutic efficacy.

## Discussion

 PXD101 effectively inhibited proliferation of eight thyroid cancer cell lines originating from four major histological types. Among seven thyroid cancer lines, ATC was more sensitive than follicular and well differentiated cancers. These findings suggest that ATC likely depends on HDACs more than the other cancer types. TT also has a low Dm, implying HDACs are important for parafollicular thyroid cancer cells. PXD101 inhibits a broad spectrum of HDACs, including class I, IIa and IIb that prevents to conclude which HDAC is more important in the survival of thyroid cancers.

 One therapeutic mechanism of HDAC inhibitors in treating malignancy is through the induction of apoptosis. PXD101 caused apoptotic effects in a dose- and time-dependent manner in BHP7-13, WRO82-1 and 8505C, suggesting that this mechanism accounts for therapeutic efficacy of PXD101.

Prior reports show HDAC inhibitors reduce thioredoxin activity, accumulate ROS and lead to apoptosis in transformed cells, but not in normal cells [6,32]. Therefore, ROS accumulation in malignant cells may be a mechanism of cancer-specificity cytotoxicity of HDAC inhibitors. In this study, PXD101 accumulated ROS in a dose-dependent fashion in WRO82-1 and 8505C, but not in the resistant cell line BHP7-13. These observations are consistent with this mechanism of susceptibility to HDAC inhibitors.

 RAS/RAF/ERK and PI3K/AKT/mTOR signaling pathways are important in thyroid cancer tumorigenesis, progression and survival [4,33]. The interruption of these signaling pathways is one strategy to treat thyroid malignancy [29,34]. In this study, PXD101 inhibited these pathways in the sensitive cell line 8505C, but not in WRO82-1 and BHP7-13. The data imply that the ability of PXD101 to inhibit RAS/RAF/ERK and PI3K/AKT/mTOR pathways may confer sensitivity.

p-H2AX is a classic marker of DSBs, a serious type of DNA damage [35]. PXD101 significantly induced p-H2AX in three thyroid cancer cell lines, supporting DSBs as one mechanism accounting for the cytotoxicity of PXD101. We show that PXD101 decreases DSBs repair proteins in the NHEJ (KU70 and KU80) and HR (RAD51) pathways. For NHEJ, the KU70-KU80 heterodimer recognizes DSBs, and recruits a DNA protein kinase complex. The decreased expression of KU70 or KU80 can impair the NHEJ repair pathway [27,36]. For HR, RAD51 binds to the resected end of single-stranded DNA (RAD51-ssDNA nucleoprotein filament), allowing for synthesis-dependent strand annealing for DSB repair [37]. An analysis of the extent of the contribution of individual HR proteins to DNA repair and found that depletion of RAD51 induces the most significant HR defect [37,38]. The SSA pathway is an alternative mechanism for DSBs repair when NHEJ and HR are defective [27,39]. PXD101 increased RAD52 and ERCC1, suggesting the SSA pathway was activated. However, when p-H2AX is increased, activation of SSA seems insufficient to repair DSBs [40]. In addition to PXD101, other HDAC inhibitors are able to repress DSB repair proteins [41-43]. Some HDACs are responsible for DNA repair; HDAC1 and 2 promote NHEJ, and HDAC 9 and 10 are required for HR [44,45].

 PXD101 treatment significantly repressed 8505C tumor growth during the study period. PXD101 transiently increased acetylation of histone H4 that is consistent with prior report [17]. Increase of p-H2AX suggests PXD101 induced DSBs in 8505C xenografts. The anti-tumor effect of PXD101 may be through apoptosis and inhibition of proliferation since caspase-3 and PCNA were decreased. No significant weight loss or toxicity observed in this study, suggesting a favorable safety profile. We also examined the therapeutic effects of PXD101 in mice bearing TT tumors. Daily intraperitoneal injection of PXD101 (40 mg/kg) for 5 doses per week failed to repress the growth of TT xenografts (data not shown). It is possible that a more intensive PXD101 treatment regimen may affect the growth of TT xenografts.

 ATC is the most aggressive thyroid cancer and is typically fatal, with a 1-year survival rate of just 20% [28]. Novel therapies are needed to improve dismal outcomes. We found that the combination of PXD101 with doxorubicin and PXD101 with paclitaxel had synergistic effects against four ATC cell lines. Prior report shows heterogeneity of cancer cells appears even in a single tumor [46]. Therefore, the combination regimen with synergistic effects against multiple ATC cell lines that have varied genetic background may be of clinical relevance. Doxorubicin inhibits topoisomerase II and causes breaks in genomic DNA [47]. PXD101 inhibited double-stranded DNA repair machinery that may enhance the cytotoxicity of doxorubicin. Paclitaxel has shown a 53% response rate against ATC in a phase II trial [48]. The favorable combination effects of PXD101 with paclitaxel support consideration of use of this therapy in patients with ATC.

 Recently, Chan et al. also reported that PXD101 retards the growth of PTC xenograft tumors [49]. PXD101 therefore has an ability to inhibit the growth of both well-differentiated and undifferentiated thyroid cancer *in vivo*. These data strengthen the possibility that PXD101 can be used to treat patients with this fatal disease. In contrast to our findings, PXD101 consistently repressed p-AKT (Ser473) and p-ERK in the prior study. One potential explanation of this inconsistency between two studies is the very high dose of PXD101 (≥ 20-fold) that was applied in previous study as compared with our current study. Such high doses of PXD101 are more likely to repress p-AKT and p-ERK. Our study additionally demonstrates that multiple molecular events induced by PXD101 may cause cytotoxicity, and shows the efficacy of combination therapy using PXD101 with conventional chemotherapy currently in use for anaplastic thyroid cancer. Importantly, we demonstrate synergistic effects of combination PXD101 with doxorubicin and paclitaxel, suggesting likely clinical significance in treating patients with ATC.

In conclusion, PXD101 imposed significant cytotoxicity in four major histologic types of thyroid cancer. Nude mice bearing 8505C xenograft tumors demonstrated the therapeutic efficacy and safety profiles of PXD101. Importantly, PXD101 synergistically improves the therapeutic effect of doxorubicin and paclitaxel against four ATC cell lines. These favorable data support the design of future clinical trials studying the utility of PXD101 as an agent to treat patients with advanced thyroid cancer.

## Materials and Methods

### Cell lines

Eight well-established thyroid cancer cell lines were studied, including a papillary (BHP7-13), a follicular (WRO82-1), a follicular undifferentiated (FRO81-2), four anaplastic (8305C, 8505C, KAT18, KAT4C) and a medullary (TT) human thyroid cancer [29,50-52]. All cell lines except KAT4C were authenticated using DNA (short tandem repeats) profiling and stored in liquid nitrogen before use [29,53]. BHP7-13, WRO82-1, FR081-2, KAT4C and KAT18 were maintained in RPMI 1640 with sodium bicarbonate (2.0 g/L). 8505C and 8305C were maintained in MEM with sodium pyruvate (1 mmol/L) and sodium bicarbonate (2.2 g/L). TT was maintained in F12K. All media contained 10% FCS, 100,000 units/L penicillin and 100 mg/L streptomycin. All cells were maintained in a 5% CO2 humidified incubator at 37 °C.

### Pharmacologic agents

PXD101 is a pan-HDAC inhibitor described previously [17], and was obtained from Selleck Chemicals. PXD101 was dissolved in DMSO (10 mmol/L for *in vitro* and 100 mg/ml for *in vivo* studies; Sigma) and stored at -30 °C until experiments. For *in vivo* studies, PXD101 was further diluted with PBS to a final concentration of 8.6 mg/ml. Doxorubicin, paclitaxel and docetaxel were obtained from Sigma Chemical Co. Doxorubicin (5 mmol/L) was dissolved in PBS, paclitaxel (320 μmol/L) and docetaxel (640 μmol/L) were dissolved in DMSO and stored at -30 °C until use.

### Antibodies

Antibodies targeting acetyl. histone H3 and histone H4 were from EMD Millipore. Acetyl. tubulin and α-tubulin antibodies were from Sigma. Caspase-3, p-ERK1/2 (Thr202/Tyr204), p-AKT (Ser473), p-4E-BP1 (Thr37/46), p-S6 ribosomal protein (Ser235/236), p-H2AX (Ser139), KU70, KU80, RAD52, ERCC1 and PCNA antibodies were from Cell Signaling. RAD51 antibody was from Invitrogen.

### Cytotoxicity assays

Cells were plated at 2 x 10^3^ to 2 x 10^4^ cells per well in 24-well plates in 1 mL media. After overnight incubation, PXD101 or vehicle were added at the indicated concentration. Six serial 1:1 dilutions of PXD101 were tested starting at 10 μmol/L over a 4-day treatment course. Cytotoxicity was determined on day 4. Cells were washed with PBS and lysed with Triton X-100 (1.35%, Sigma) to release intracellular lactate dehydrogenase (LDH), which was quantified with a Cytotox 96 kit (Progmega) at 490 nM by spectrophotometry (BT-MQX200R, Bio-Tek Instruments). Each experiment was performed in triplicate and results are shown as the percentage of surviving cells determined by comparing the LDH of each sample relative to control samples which are considered 100% viable. Dm on day 4 were calculated for each cell line using CompuSyn software [30,31].

For combination therapy experiments, ATC cells were treated with PXD101 and a chemotherapeutic drug (doxorubicin, paclitaxel or docetaxel) at a fixed dose ratio. Cells were incubated with vehicle, PXD101, chemotherapeutic agent, or both simultaneously for a 4-day course and cytotoxicity was measured. Six to seven serial 1:1 dilutions were examined at the following starting doses for 8305C, 8505C, KAT18 and KAT4C: PXD101 at 2.4, 3.6, 3.6 and 4.4 μmol/L, doxorubicin at 0.64, 0.16, 0.22, 0.27 μmol/L, paclitaxel at 50.8, 22.4, 17.6 and 13.2 nmol/L, docetaxel at 34.4, 6.8, 5.2 and 7.6 nmol/L, respectively. The doses chosen were based on the Dm determined in this and previous studies [29].

### Western blots

Cells were plated at 1 x 10^6^ cells in 10-cm Petri dishes in 10 mL media overnight and treated with PXD101 or vehicle for 48 hours. Cell pellets were dissolved in radio-immunoprecipitation assay buffer and protease inhibitor cocktail, vortexed and clarified by centrifugation. Total protein (20–50 μg) was electrophoresed on 10–12% Tris-HCl gels, transferred to polyvinylidene difluoride membranes, blocked, and exposed to primary antibodies followed by a secondary antibody conjugated to horseradish peroxidase. Signals were developed using an enhanced chemiluminescence kit (Bionovas Biotechnology). α-tubulin served as loading control.

### Apoptosis and ROS assessment

Cells were plated at 1 x 10^5^ cells per well in 6-well plates in 2 mL media overnight and treated with placebo or PXD101 at indicated doses for 48 and 72 hours. Floating cells and trypsinized adherent cells were collected, washed with PBS, fixed with cold 70% ethanol and incubated with RNase A (100 µg/mL; Sigma) and propidium iodide (5 µg/mL; Sigma) at 37°C for 15 min. Apoptotic sub-G1 cells were detected by DNA content using flow cytometry (BD FACScalibur Flow Cytometer, BD Biosciences).

Cells were plated at 1 x 10^5^ cells per well in 6-well plates in 2 mL media for 24 hours and treated with placebo or PXD101 at indicated doses for 16 hours. Trypsinized adherent cells were collected and incubated with 10 μmol /L DCFH-DA (Sigma) at 37°C in the dark for 30 minutes. Cells were centrifuged, resuspended with PBS and probed intracellular DCFH-DA fluorescence to measure ROS by using flow cytometry (BD FACScalibur Flow Cytometer, BD Biosciences). The fluorescence intensities of untreated and treated cells were gained and mean values were calculated. Each condition was performed in triplicate.

### Flank xenograft tumor therapy

Six-week-old athymic female nude mice (National Laboratory Animal Center, Taiwan) were anesthetized with intraperitoneal injection of ketamine hydrochloride (90 mg/kg; Nang Kuang Pharmaceutical Co.) and xylazine hydrochloride (9 mg/kg; Bayer) before implantation of thyroid cancer cells. 8505C flank tumors were established by injecting 1 x 10^6^ cells in 100 μL PBS into the subcutaneous flanks of nude mice. When tumors reached 6.6 mm in mean diameter, mice (*n* = 13-14 per group) were treated with PXD101 (40 mg/kg) or placebo by intraperitoneal injection daily of 5 days per week. Tumor dimensions were serially measured with electronic calipers, and the volumes were calculated by the formula a^2^ x b x 0.4, where a represents the smallest diameter and b is the perpendicular diameter. The animals were followed for body weight as a marker of toxicity.

Tumor levels of acetyl. histone H3, acetyl. histone H4, p-H2AX (Ser139), p-AKT (Ser473), RAD51, caspase-3 and PCNA were evaluated in mice treated with a single dose of PXD101 (40 mg/kg). At indicated periods, animals were sacrificed using carbon dioxide, tumors were harvested, mixed with protein extraction buffer (GE Healthcare), homogenized and sonicated on ice. After centrifugation, clarified supernatants were aliquoted and stored at -80 °C until western blot was performed.

This study was performed in accordance with the recommendations in the Guide for the Care and Use of Laboratory Animals of Chang Gung Memorial Hospital and the protocol was approved by the Committee of Laboratory Animal Center at Chang Gung Memorial Hospital, Linkou.

### Quantitative analysis of drug interactions and statistical analyses

Interactions between PXD101 and the chemotherapeutic drugs for each cell line were determined by calculating the CI of Chou-Talalay equation [30]. The dose-effect analysis was produced using CompuSyn software [31], following the dose and effect data entries. DRI was also determined. DRI represents the fold dose-reduction permitted by the combination, for a given effect level, when compared with each drug alone. Comparisons were performed when appropriate using two sided Student’s t test (Excel, Microsoft). Statistical significance is considered if P < 0.05. Results were expressed as the mean ± SE.

## Supporting Information

Figure S1 **The combination therapy of PXD101 and chemotherapeutic agents enhances cytotoxicity against ATC.** The interactions between PXD101 and chemotherapeutic agents (doxorubicin, paclitaxel or docetaxel) after a 4-day treatment in four ATC cancer lines were evaluated using LDH assays. The combination of PXD101 and chemotherapeutic agents revealed favorable therapeutic effects in all cell lines.
(TIF)

Figure S2 **PXD101 represses RAD51 rapidly and transiently *invivo*.** PXD101 greatly reduced RAD51 to less than 21% protein remaining at 3 hours, followed by overexpression of RAD51 from 6 to 24 hours.
(TIF)
