# Supplementary figures and images for: Utility of a Histone Deacetylase Inhibitor (PXD101) for Thyroid Cancer Treatment
Source: PLoS One. 2013 Oct 14;8(10):e77684. doi: 10.1371/journal.pone.0077684 (PMC3796495; doi:10.1371/journal.pone.0077684)

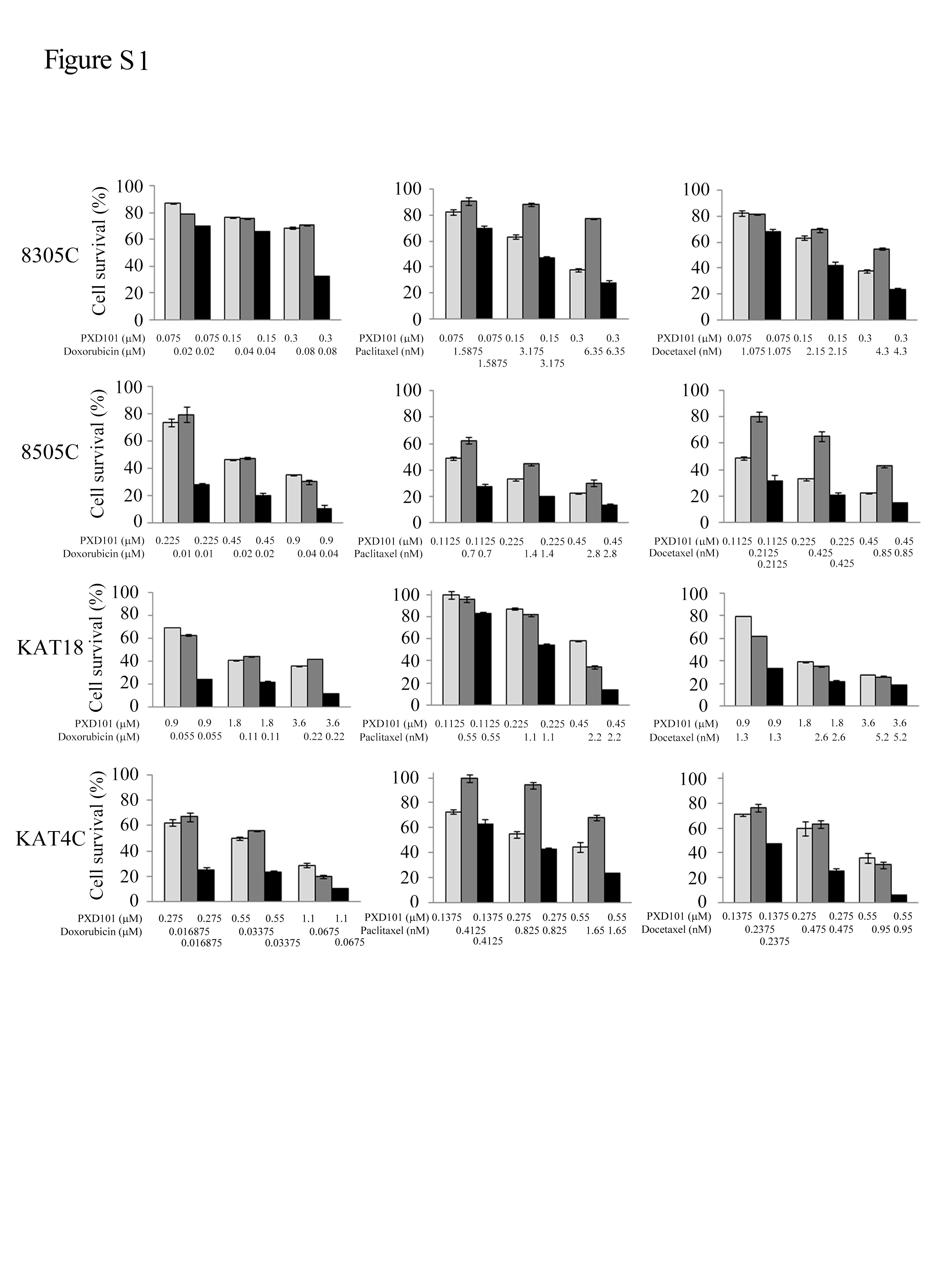

Supplement: Figure S1 — The combination therapy of PXD101 and chemotherapeutic agents enhances cytotoxicity against ATC. The interactions between PXD101 and chemotherapeutic agents (doxorubicin, paclitaxel or docetaxel) after a 4-day treatment in four ATC cancer lines were evaluated using LDH assays. The combination of PXD101 and chemotherapeutic agents revealed favorable therapeutic effects in all cell lines. (TIF) [file pone.0077684.s001.tif]

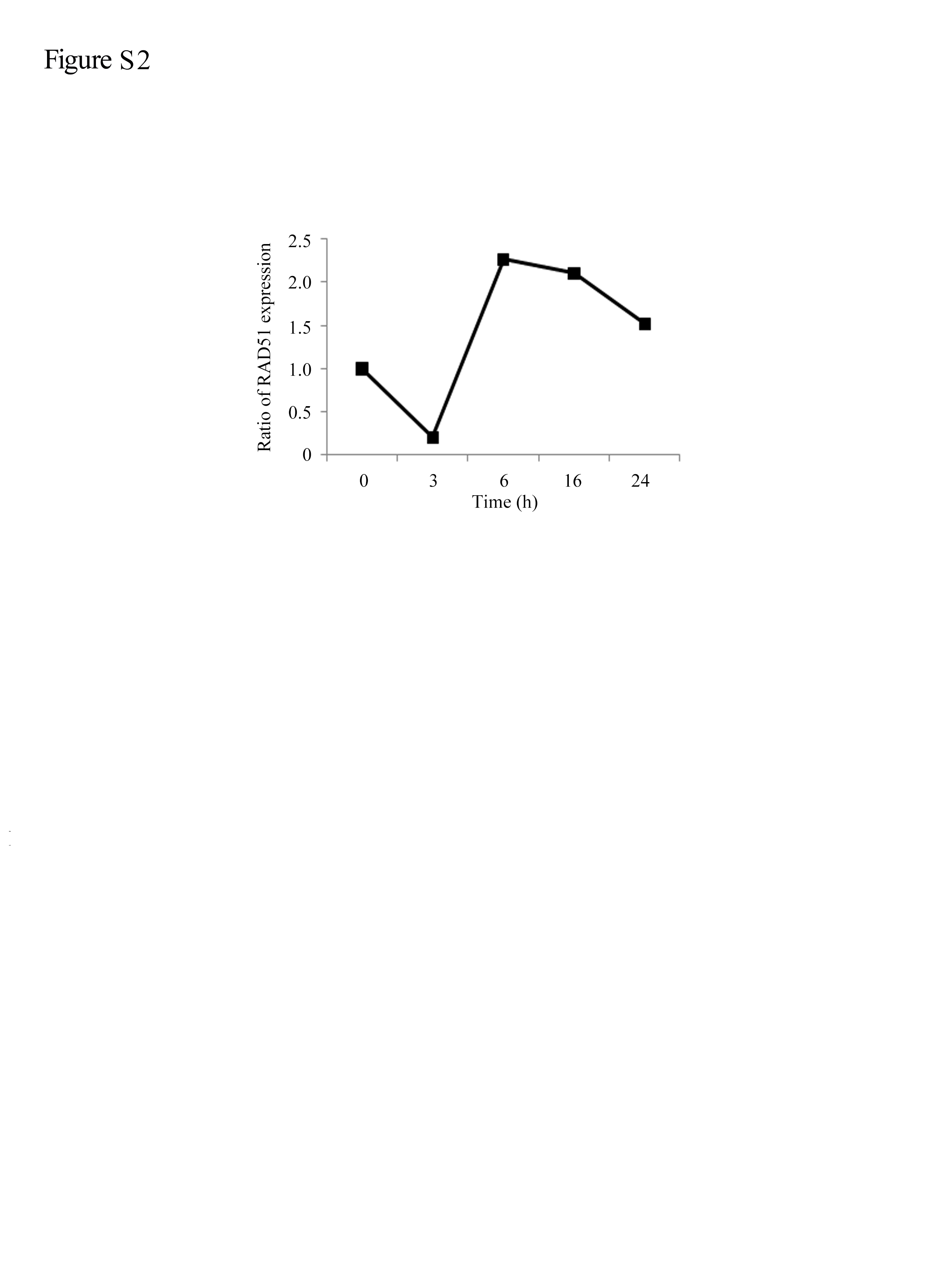

Supplement: Figure S2 — PXD101 represses RAD51 rapidly and transiently invivo. PXD101 greatly reduced RAD51 to less than 21% protein remaining at 3 hours, followed by overexpression of RAD51 from 6 to 24 hours. (TIF) [file pone.0077684.s002.tif]
